# Apolipoprotein C-III in patients with systemic lupus erythematosus

**DOI:** 10.1186/s13075-022-02793-y

**Published:** 2022-05-10

**Authors:** Candelaria Martín-González, Carmen Ferrer-Moure, Juan Carlos Quevedo-Abeledo, Antonia de Vera-González, Alejandra González-Delgado, Julio Sánchez-Martín, Miguel Á. González-Gay, Iván Ferraz-Amaro

**Affiliations:** 1 Division of Internal Medicine, Hospital Universitario de Canarias, Tenerife, Spain; 2 Division of Central Laboratory, Hospital Universitario de Canarias, Tenerife, Spain; 3 Division of Rheumatology, Hospital Doctor Negrín, Las Palmas de Gran Canaria, Spain; 4 Epidemiology, Genetics and Atherosclerosis Research Group On Systemic Inflammatory Diseases, Hospital Universitario Marqués de Valdecilla, IDIVAL, Santander, Spain; 5 Division of Rheumatology, Hospital Universitario Marqués de Valdecilla, Universidad de Cantabria, Santander, Spain; 6 Cardiovascular Pathophysiology and Genomics Research Unit, School of Physiology, Faculty of Health Sciences, University of the Witwatersrand, Johannesburg, South Africa; 7 Division of Rheumatology, Hospital Universitario de Canarias, Tenerife, Spain

**Keywords:** Systemic lupus erythematosus, Apolipoprotein C3

## Abstract

**Background:**

Systemic lupus erythematosus (SLE) has been associated with atherosclerotic cardiovascular disease (CV) and an altered lipid profile. High levels of apolipoprotein C-III (ApoC3) are associated with elevated triglyceride levels and an increased risk of CV. In the present study, we aimed to study circulating ApoC3 in patients with SLE and describe its relationship with the manifestations of the disease.

**Methods:**

This is a cross-sectional study that included 186 patients with SLE. Disease-related data, CV comorbidity, full lipid profile, and serum levels of ApoC3 were assessed. A multivariable regression analysis was performed to study how ApoC3 was related to SLE features.

**Results:**

Classic CV risk factors were significantly and strongly associated with circulating ApoC3. After a fully multivariable analysis that included classic CV risk factors and lipid profile molecules, SLICC damage (beta coef. 0.10 [95% *CI* 0.02–0.19] mg/dl, 0.020) and Katz severity (beta coef. 0.11 [95% *CI* 0.03–0.19] mg/dl, *p* = 0.011) indices and SLEDAI activity score (beta coef. 0.05 [95% *CI* 0.05–0.08] mg/dl, *p* = 0.004) were all independently associated with higher levels of circulating ApoC3.

**Conclusion:**

Among SLE patients, disease activity, severity, and disease damage are independently associated with higher ApoC3 serum levels.

**Supplementary Information:**

The online version contains supplementary material available at 10.1186/s13075-022-02793-y.

## Background

Systemic lupus erythematosus (SLE) has been associated with increased cardiovascular (CV) disease [1] and altered lipid profile [2]. Although the exact mechanisms by which this occurs are unknown, it is believed that the inflammation that accompanies the disease is responsible for dyslipidemia and the increased CV risk that occurs in patients with SLE [3]. Importantly, despite their heightened risk, patients with SLE are often undertreated for known causative agents and exacerbators of atherosclerotic CV disease [4].

Apolipoprotein C-III (ApoC3) is a small protein that resides on the surface of very-low-density lipoproteins, low-density lipoproteins, chylomicrons, and high-density lipoproteins. ApoC3 is a major regulator of lipolysis, as it inhibits lipoprotein lipase, the enzyme that hydrolyzes triacylglycerols in triacylglycerol-rich lipoproteins, which generates smaller triacylglycerol-depleted remnant lipoproteins. A growing body of evidence suggests that ApoC3 is a multifunctional protein that not only regulates the metabolism of triglycerides; it is also an important regulator of endothelial function. Its ability to induce endothelial dysfunction would link hyperlipidemia with atherosclerosis and is a mechanism by which lipids can increase inflammatory responses associated with the development of atherosclerotic lesions, thus increasing the risk of atherosclerotic CV disease [5]. Accordingly, high levels of ApoC3 have been found to be associated with elevated triglyceride levels and an increased risk of atherosclerotic CV disease, and low levels of ApoC3 are associated with lower triglyceride levels and reduced CV risk [5].

ApoC3 serum levels have been partially evaluated before in SLE patients. For this reason, in the present work, we have studied ApoC3 in a large series of patients with SLE. Our objective was to determine ApoC3 serum levels in SLE patients and how they are related to the manifestations and comorbidities of the disease.

## Material and methods

### Study participants

This was a cross-sectional study that included 186 patients with SLE. All SLE patients were 18 years old or older, had a clinical diagnosis of SLE, and fulfilled ≥ 4 American College of Rheumatology (ACR) classification criteria for SLE [6]. They had been diagnosed by rheumatologists and were periodically followed up at rheumatology outpatient clinics. Apart from the possible use of statins, the subjects included in the study were required to have no conditions or pharmacological treatment that could influence lipids and were not taking any other lipid-lowering medications. Consequently, patients taking statins were allowed to participate in the study. Patients taking prednisone, at an equivalent dose ≤ 10 mg/day, were allowed to participate, as glucocorticoids are often used in the treatment of SLE. Furthermore, since diabetic dyslipidemia has a characteristic pattern, patients with diabetes mellitus were not included in this study. Besides, patients were excluded if they had a history of myocardial infarction, angina, stroke, a glomerular filtration rate < 60 ml/min/1.73 m^2^, a history of cancer, or any other chronic disease, or evidence of active infection. Research was carried out in compliance with the Declaration of Helsinki. The study protocol was approved by the Institutional Review Committee at Hospital Universitario de Canarias and at Hospital Universitario Doctor Negrín (both in Spain), and all subjects provided informed written consent (Approval Number 2015_84).

### Data collection and laboratory assessments

Individuals included in the study completed a CV risk factor and medication use questionnaire and underwent a physical examination. Weight, height, body mass index, abdominal circumference, and systolic and diastolic blood pressure (measured with the participant in a supine position) were assessed under standardized conditions. Information regarding smoking status (current smokers) and hypertension treatment was obtained from the questionnaire. Medical records were reviewed to ascertain specific diagnoses and medications. SLE disease activity and damage were assessed using the Systemic Lupus Erythematosus Disease Activity Index-2000 (SLEDAI-2 K) [7] and the SLICC/ACR Damage Index (SDI) [8], respectively. For the purpose of the present study, the SLEDAI-2 K index was broken down into none (0 points), mild (1–5 points), moderate (6–10 points), high (11–19), and very high activity (> 20) as previously described [9]. Disease severity was measured as well, using the Katz index [10]. An ELISA kit was used for the detection of ApoC3 (Elabscience, USA). With this kit, no significant cross-reactivity or interference is observed between human ApoC3 and its analogs. Both intra- and inter-coefficients of variability are < 10% for this assay. Cholesterol, triglycerides, and HDL cholesterol were measured using the enzymatic colorimetric assay. LDL cholesterol was calculated using Friedewald’s formula, and the atherogenic index has been estimated through Castelli’s formula (total cholesterol/HDL cholesterol).

### Statistical analysis

Demographic and clinical characteristics in patients with SLE and controls were described as mean ± standard deviation (SD) or percentages for categorical variables. For non-normally distributed continuous variables, data were expressed as median and interquartile range (IQR). Relation of features of the disease with circulating ApoC3 was assessed through univariable linear regression analysis. Furthermore, the association of different SLE scores with ApoC3 was analyzed using multivariable linear regression analysis. The confounders were selected from the CV risk factors that had a univariable relationship with ApoC3 with a *p* value less than 0.20 and from the different molecules of the lipid pattern with which a given disease score also had a *p* inferior to 0.20. Because of collinearity, lipid pattern variables derived from a formula were excluded from the regression models (LDL cholesterol, LDL:HDL ratio, non-HDL cholesterol, apolipoprotein B:apolipoprotein A ratio, and atherogenic). All the analyses used a 5% two-sided significance level and were performed using Stata software, version 17/SE (StataCorp, College Station, TX, USA). *p*-values < 0.05 were considered statistically significant.

## Results

### Demographics and disease-related data of systemic lupus erythematosus patients

Demographic and disease-related characteristics of the 186 patients with SLE included in this study are shown in Table 1. Most of them were women (95%), and the mean age ± SD was 50 ± 11 years. The body mass index of the participants was 27 ± 5 kg/m^2^, and the average abdominal circumference was 92 ± 13 cm. Classic CV risk factors were not uncommon. For example, 17% of the patients were current smokers, 28% had hypertension, and 26% were obese. Likewise, 24% of the patients were taking statins (mostly simvastatin 20 mg/day and atorvastatin 10 mg/day), and 26% and 35% were under aspirin or antihypertensive treatment, respectively. The lipid profile values of patients with SLE are shown in Table 1. Cholesterol was 200 ± 38 mg/dl, and LDL and HDL cholesterol were, respectively, 111 ± 29 and 63 ± 21 mg/dl. Apolipoprotein C3 median serum levels in SLE patients were 1.75 (*IQR* 1.24–2.59) mg/dl.

**Table 1:** Characteristics of the SLE patients

	SLE patients
	(*n* = 186)
Age, years	50 ± 11
Women, *n* (%)	177 (95)
Body mass index, kg/m^2^	27 ± 5
Abdominal circumference, cm	92 ± 13
Systolic blood pressure, mmHg	128 ± 20
Diastolic blood pressure, mmHg	84 ± 52
*Cardiovascular comorbidity*	
Smoking, *n* (%)	31 (17)
Diabetes, *n* (%)	0 (0)
Hypertension, *n* (%)	70 (28)
Obesity, *n* (%)	48 (26)
Statins, *n* (%)	45 (24)
Aspirin, *n* (%)	48 (26)
Antihypertensive treatment, *n* (%)	65 (35)
*Analytical and lipid profile data*	
CRP, mg/dl	1.9 (0.9–4.9)
Cholesterol, mg/dl	200 ± 38
Triglycerides, mg/dl	127 ± 81
HDL cholesterol, mg/dl	63 ± 21
LDL cholesterol, mg/dl	111 ± 29
LDL:HDL cholesterol ratio	1.92 ± 0.74
Non-HDL cholesterol, mg/dl	198 ± 38
Apolipoprotein A1, mg/dl	179 ± 37
Apolipoprotein B, mg/dl	96 ± 23
Apo B:A1 ratio	0.55 ± 0.16
Lipoprotein (a), mg/dl	37 (13–106)
Atherogenic index	3.4 ± 1.1
Apolipoprotein C3, mg/dl	1.75 (1.24–2.59)
*SLE-related data*	
Disease duration, years	17 ± 9
SLICC	1 (0–2)
SLICC ≥ 1, *n* (%)	136 (73)
Katz index	2 (1–3)
SLEDAI	2 (0–5)
SLEDAI categories, *n* (%)	
No activity, *n* (%)	73 (39)
Mild or moderate, *n* (%)	90 (48)
High or very high, *n* (%)	13 (7)
Auto-antibody profile	
Anti-DNA positive, *n* (%)	96 (52)
ENA positive, *n* (%)	63 (34)
Anti-Ro, *n* (%)	61 (33)
Anti-La, *n* (%)	30 (16)
Anti-RNP, *n* (%)	47 (25)
Anti-Sm, *n* (%)	21 (11)
Any antiphospholipid autoantibodies, *n* (%)	
Lupus anticoagulant, *n* (%)	36 (19)
ACA IgM, *n* (%)	20 (11)
ACA IgG, *n* (%)	30 (16)
Anti-beta2 glycoprotein IgM, *n* (%)	12 (6)
Anti-beta2 glycoprotein IgG, *n* (%)	22 (12)
C3, mg/dl	143 ± 52
C4, mg/dl	27 ± 14
Current prednisone, *n* (%)	95 (51)
Prednisone, mg/day	5 (5–7.5)
DMARDs, *n* (%)	144 (77)
Hydroxychloroquine, *n* (%)	126 (68)
Hydroxychloroquine, mg/day	200 (200–300)
Methotrexate, *n* (%)	21 (11)
Mycophenolate mofetil, *n* (%)	15 (8)
Azathioprine, *n* (%)	25 (13)
Rituximab, *n* (%)	6 (3)
Belimumab, *n* (%)	3 (2)

Data represent mean ± SD or median (interquartile range) when data were not normally distributed. SLEDAI categories were defined as 0, no activity; 1–5, mild; 6–10, moderate; > 10 activity. DMARDs refer to the use of at least one of hydroxychloroquine, methotrexate, azathioprine, or mycophenolate

*BMI* body mass index, *C3 C4* complement, *CRP* C-reactive protein, *LDL* low-density lipoprotein, *DMARD* disease-modifying antirheumatic drug, *ACA* anticardiolipin, *HDL* high-density lipoprotein, *ANA* antinuclear antibodies, *ENA* extractible nuclear antibodies, *SLEDAI* Systemic Lupus Erythematosus Disease Activity Index, *SLICC* Systemic Lupus International Collaborating Clinics/American Colleague of Rheumatology Damage Index

Disease duration was 17 ± 9 years. Most of the patients with SLE were in the categories of no activity (39%) or mild-moderate activity (48%) as shown by the SLEDAI scores. SLICC and Katz indexes were 1 (*IQR* 0–2) and 2 (*IQR* 1–3), respectively. Seventy-three percent of the patients had a SLICC/ACR DI score equal to or higher than 1. Half of the patients (51%) were taking prednisone. The median daily dose of prednisone was 5 mg/day (IQR 5–7.5 mg). At the time of recruitment, 52% patients were found to be positive for anti-DNA, and 34% were positive for ENA, with anti-Ro being the antibody most frequently found (33%). Disease-modifying antirheumatic drug use was reported in 77% of the patients and 68% were taking hydroxychloroquine when the study was performed (median dose 200 [*IQR* 200–300] mg/day). Other less used disease-modifying antirheumatic drugs were methotrexate (11%) and azathioprine (13%). Additional information of SLE-related data is shown in Table 1.

### Univariable analysis of the relationship of demographic and disease-related data with serum levels of apolipoprotein C3

The classic CV risk factors were generally significantly and strongly associated with circulating ApoC3. In this sense, being hypertensive and obese or having a superior body mass index and abdominal circumference was all associated with higher serum levels of ApoC3. Additionally, patients taking statins or antihypertensive treatment had elevated serum ApoC3 levels compared to those not taking these therapies (Table 2). Also, while CRP, triglycerides, and atherogenic index were significantly and positively associated with ApoC3, HDL cholesterol and apolipoprotein A1 were negatively related to it.

**Table 2:** Demographics and disease-related data relation to apolipoprotein C3

	Apo C3, mg/dl	
	Beta coef. (95% *CI*), *p*	
Age, years	**0.04 (0.02–0.05)**	** < 0.001**
Women	0.19 (− 0.70–0.91)	0.80
Body mass index, kg/m^2^	**0.04 (0.02–0.08)**	**0.004**
Abdominal circumference, cm	**0.02 (0.01–0.04)**	** < 0.001**
Systolic blood pressure, mmHg	**0.01 (0.00–0.02)**	**0.003**
Diastolic blood pressure, mmHg	− 0.00 (− 0.00–0.00)	0.63
*Cardiovascular comorbidity*		
Smoking	0.11 (− 0.70–0.91)	0.80
Diabetes	-	
Hypertension	**0.73 (0.40–1.07)**	** < 0.001**
Obesity	**0.51 (0.14–0.88)**	**0.008**
Statins	**0.57 (0.18–0.95)**	**0.004**
Aspirin	0.01 (− 0.37–0.40)	0.94
Antihypertensive treatment	**0.72 (0.38–1.06)**	** < 0.001**
*Analytical and lipid profile*		
CRP, mg/dl	**0.01 (**− **0.00–0.02)**	**0.055**
Cholesterol × 10, mg/dl	− 0.01 (− 0.06–0.02)	0.39
Triglycerides × 10, mg/dl	**0.03 (0.01–0.06)**	**0.002**
HDL cholesterol × 10, mg/dl	** − 0.16 (− 0.25 to − 0.07)**	** < 0.001**
LDL cholesterol × 10, mg/dl	− 0.01 (− 0.07–0.04)	0.72
LDL:HDL cholesterol ratio	**0.30 (− 0.08–0.53)**	**0.009**
Non-HDL cholesterol × 10, mg/dl	− 0.02 (− 0.06–0.02)	0.36
Apolipoprotein A1 × 10, mg/dl	** − 0.05 (− 010– -0.01)**	**0.026**
Apolipoprotein B × 10, mg/dl	− 0.00 (− 0.07–0.06)	0.94
Apo B: A1 ratio	0.70 (− 0.33–1.72)	0.18
Lipoprotein (a) × 10, mg/dl	− 0.00 (− 0.02–0.01)	0.60
Atherogenic index	**0.24 (0.08–0.39)**	**0.003**
*SLE-related data*		
Disease duration, years	**0.02 (0.00–0.04)**	**0.048**
SLICC	**0.14 (0.06–0.23)**	**0.001**
SLICC ≥ 1	**0.46 (0.07–0.85)**	**0.020**
Katz index	**0.10 (0.01–0.19)**	**0.030**
SLEDAI	0.02 (− 0.01–0.06)	0.19
SLEDAI categories		
No activity	ref	
Mild and moderate	**0.39 (0.03–0.75)**	**0.035**
High or very high	**0.69 (0.02–1.37)**	**0.045**
Auto-antibody profile		
Anti-DNA positive	− 0.11 (− 0.56–0.34)	0.63
ENA positive	− 0.12 (− 0.74–0.50)	0.70
Anti-Ro	− 0.17 (− 0.56–0.23)	0.40
Anti-La	− 0.27 (− 0.75–0.21)	0.26
Anti-RNP	0.09 (− 0.31–0.49)	0.66
Anti-Sm	− 0.34 (− 0.87–0.18)	0.19
Any antiphospholipid autoantibodies, *n* (%)		
Lupus anticoagulant	0.16 (− 0.28–0.61)	0.47
ACA IgM	− 0.03 (− 0.59–0.54)	0.93
ACA IgG	0.22 (− 0.25–0.70)	0.35
Anti-beta2 glycoprotein IgM	− 0.03 (− 0.73–0.67)	0.93
Anti-beta2 glycoprotein IgG	0.40 (− 0.14–0.93)	0.14
C3 × 10, mg/dl	− 0.00 (− 0.7–0.05)	0.77
C4 × 10, mg/dl	0.09 (− 0.15–0.33)	0.47
Current prednisone	0.23 (− 0.11–0.56)	0.19
Prednisone, mg/day	**0.09 (0.01–0.16)**	**0.023**
DMARDs	− 0.36 (− 0.77–0.04)	0.080
Hydroxychloroquine	− 0.33 (− 0.69–0.03)	0.072
Hydroxychloroquine, mg/day × 10	− 0.01 (− 0.03–0.006)	0.18
Methotrexate	− 0.03 (− 0..56–0..50)	0.92
Mycophenolate mofetil	− 0.23 (− 0.83–0.37)	0.45
Azathioprine	0.37 (− 0.12–0.86)	0.13
Rituximab	− 0.47 (− 1.39–0.46)	0.32
Belimumab	0.29 (− 1.01–1.59)	0.66

Beta coefficients consider apolipoprotein C3 as the dependent variable. Significant *p* values are depicted in bold

*BMI* body mass index, *C3 C4* complement, *CRP* C-reactive protein, *LDL* low-density lipoprotein, *DMARD* disease-modifying antirheumatic drug, *ACA* anticardiolipin, *HDL* high-density lipoprotein, *ANA* antinuclear antibodies, *ENA* extractible nuclear antibodies, *SLEDAI* Systemic Lupus Erythematosus Disease Activity Index, *SLICC* Systemic Lupus International Collaborating Clinics/American Colleague of Rheumatology Damage Index

Disease duration (beta coef. 0.02 [95% *CI* 0.00–0.04], mg/dl, *p* = 0.048) was positively related to ApoC3. Remarkably, SLE scores of disease activity, severity, and damage were all associated with higher serum levels of ApoC3. In this sense, SLICC score, both continuous (beta coef. 0.14 [95% *CI* 0.06–0.23], *p* = 0.001) and binary — equal or higher than 1 — (beta coef. 0.46 [95% *CI* 0.07–0.85], *p* = 0.020), was related to a superior circulating ApoC3. Similarly, the Katz index was significantly and positively associated with higher serum levels of ApoC3 (beta coef. 0.10 [95% *CI* 0.01–0.19], *p* = 0.030). Also, patients included in the mild or moderate (beta coef. 0.39 [95% *CI* 0.03–0.75], *p* = 0.035), or in the high or very high (beta coef. 0.69 [95% *CI* 0.02–1.37], *p* = 0.045) SLEDAI categories were associated with greater ApoC3 levels when compared to those included in the remission category. All the aforementioned relations were found in an univariable manner (Table 2). Other disease-related characteristics like ANA profile, antiphospholipid autoantibodies, complement serum levels, or disease-modifying antirheumatic drugs used in the disease were not associated with circulating ApoC3.

### Multivariable analysis of the relationship of SLE activity, severity, and damage scores with apolipoprotein C3

**Fig. 1:**
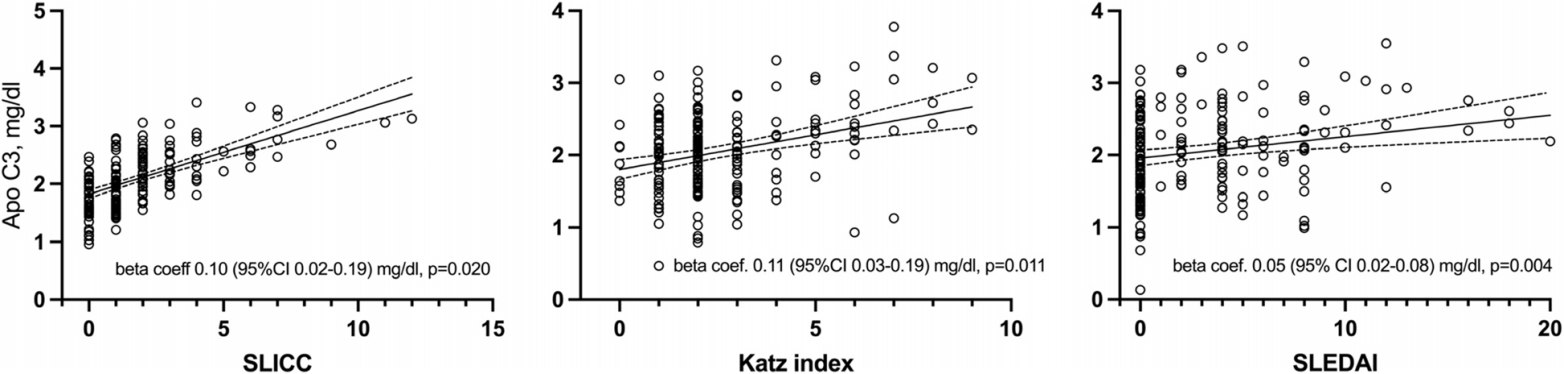
Multivariable representation of SLICC, Katz, and SLEDAI scores relationship with ApoC3

SLICC, SLEDAI, and Katz scores were not related to any of the lipid profile molecules in patients with SLE. In this sense, univariable regression analysis did not yield any significant association between those scores and cholesterol, triglycerides, apolipoproteins, lipoprotein (a), or atherogenic index (Table 3). However, SLICC (beta coef. 0.10 [95% *CI* 0.02–0.19] mg/dl, *p* = 0.020), Katz index (0.10 [95% *CI* 0.02–0.19], *p* = 0.015), and SLEDAI score (beta coef. 0.04 [95% *CI* 0.00–0.07], *p* = 0.036) were significantly associated with higher circulating ApoC3 after multivariable adjustment that included age, body mass index, systolic blood pressure, and statin use (model #1 in Table 3). The significant association remained stable when we performed another multivariable analysis adjusting for the same variables plus lipids that had a univariable relation to the disease scores inferior to 0.20 (model #2 in Table 3). A representation of this relationship is shown in Fig. 1. It was also the case when scores were stratified in different categories: SLICC (equal to or higher than 1) and SLEDAI (equal to or higher than 1; or no activity, mild or moderate, and high or very high SLEDAI) (Supplementary Table 1).

**Table 3:** Multivariable analysis of the relationship of SLE activity, severity, and damage scores with apolipoprotein C3

	SLICC	Katz index	SLEDAI
	Beta coef. (95% *CI*), *p*		
Lipid profile			
Cholesterol, mg/dl	0.3 (− 2–3), 0.84	− 0.06 (− 3–3), 0.97	1 (0–2), 0.17
Triglycerides, mg/dl	2 (− 4–8), 0.59	− 1 (− 7–6), 0.83	− 1 (− 3–2), 0.63
HDL cholesterol, mg/dl	0.1 (− 1–2), 0.88	1 (0–3), 0.12	1 (0–1), 0.11
LDL cholesterol, mg/dl	− 0.2 (− 2–2), 0.89	− 1 (− 3–1), 0.30	0.4 (0–1), 0.36
LDL:HDL cholesterol ratio	0.02 (− 0.4–0.07), 0.50	− 0.05 (− 0.11–0.01), 0.080	− 0.005 (− 0.03–0.02), 0.67
Non-HDL cholesterol, mg/dl	0.3 (− 2–3), 0.85	0.01 (− 3–3), 0.99	1 (0–2), 0.16
Apolipoprotein A1, mg/dl	1 (− 2–3), 0.66	2 (− 1–5), 0.25	1 (0–2), 0.068
Apolipoprotein B, mg/dl	− 1 (− 2–1), 0.48	− 0.2 (− 2–2), 0.82	0.2 (− 1–1), 0.59
Apo B: A1 ratio	− 0.004 (− 0.2–0.01), 0.49	− 0.01 (− 0.02–0.00), 0.22	0.0009 (− 0.01–0.00), 0.73
Lipoprotein (a), mg/dl	− 3 (− 9–4), 0.41	0.5 (-6–7), 0.89	1 (− 2–3), 0.67
Atherogenic index	0.04 (− 0.04–0.12), 0.38	− 0.06 (− 0.14–0.02), 0.16	− 0.01 (− 0.04–0.02), 0.48
Apolipoprotein C3, mg/dl			
Unadjusted	**0.14 (0.06–0.23), 0.001**	**0.10 (0.01–0.19), 0.030**	0.02 (− 0.01–0.06), 0.19
Adjusted #1	**0.10 (0.02–0.19), 0.020**	**0.10 (0.02–0.19), 0.015**	**0.04 (0.00–0.07), 0.036**
Adjusted #2		0.11 (0.03–0.19), 0.011*	0.05 (0.02–0.08), 0.004**

Beta coefficients represent the relation of SLICC, Katz, and SLEDAI score (independent variable) to each of the lipid profile molecules including ApoC3 (dependent variables). Significant *p* values are depicted in bold

*LDL* low-density lipoprotein, *HDL* high-density lipoprotein, *SLEDAI* Systemic Lupus Erythematosus Disease Activity Index, *SLICC* Systemic Lupus International Collaborating Clinics/American Colleague of Rheumatology Damage Index

^#^1 adjusted for age, BMI, systolic blood pressure, and statins

^#^2 adjusted for age, BMI, systolic blood pressure, and statins, plus any lipid profile molecule that has a univariable relation with the score inferior to 0.20

^*^Adjusted for age, BMI, systolic blood pressure, and statins + HDL cholesterol; **adjusted for age, BMI, systolic blood pressure, and statins + cholesterol, HDL cholesterol, and apolipoprotein A1

## Discussion

Our study is the first to evaluate ApoC3 in a large series of patients with SLE. According to our findings, ApoC3 is related to the classic CV risk factors that are present in these patients. However, the disease activity, severity, and damage are associated with higher circulating ApoC3 independently of the classic CV risk factors.

Previous data on ApoC3 in patients with SLE are scarce. In a report on 9 controls, 21 SLE patients, and 11 lupus nephritis patients, total ApoC3 levels were significantly increased in lupus nephritis patients compared to controls or non-renal SLE patients [11]. The authors of this study, which intended to analyze how different oxidate serum proteins vary between SLE and controls, suggested that ApoC3 could be a biomarker in the subset of patients with lupus nephritis [11]. The association between various apolipoproteins with subclinical measures of atherosclerosis (coronary artery calcification and carotid intima-media thickness) was assessed in a study that included 58 SLE patients [12]. In this study, different types of ApoC3 (Apo C-HP, Apo C3-HS, ApoC3-R) were not found to be associated with subclinical atherosclerosis. Other functions of ApoC3 in SLE were not explored in this work.

Our data indicates that ApoC3 is strongly correlated with classic CV risk factors in patients with SLE. This is in agreement with previous reports showing that elevated ApoC3 levels are associated with increased triglyceride levels and an increased risk of atherosclerotic CV disease [13]. Since patients with CV risk factors are more susceptible to develop atherosclerosis, SLE patients with elevated ApoC3 can represent a subset of patients at increased risk for CV disease. For this reason, the association found between the classic CV risk factors and the ApoC3 found in our study could probably be bidirectional, being both a consequence and a cause of the other.

In our study, the different SLE scores, although constructed with different items and expressing different aspects of the disease, were all independently related to ApoC3. The fact that all three scores, and not just one, showed the same relationship with ApoC3 reinforces our findings. Interestingly, none of them includes dyslipidemia or the use of statins among their items. Furthermore, these scores showed no association with any of the other lipid profile molecules. For these reasons, their relationship with ApoC3 cannot be attributed in any case to the fact that they modified other lipid molecules and, consequently, these other lipid molecules could also alter ApoC3. Taking into account all these findings, we believe that it is the disease per se, through its own mechanisms, that is responsible for the elevation of ApoC3 as the activity, damage, and severity of SLE increase.

Our purpose was specifically to focus on the circulating levels of ApoC3 in patients with SLE and its relationship with different manifestations of the disease. For this reason, we cannot establish whether patients with SLE have different ApoC3 levels than the general population. This may be considered as a potential limitation of our study. Besides, we acknowledge the limitation that we did not collect the total amount of hydroxychloroquine received since SLE diagnosis. For this reason, we cannot analyze the relation that cumulative hydroxychloroquine doses may have to ApoC3. Similarly, estrogens have been described to modify triglycerides or to induce dyslipidemia. Given that in our population there were 95% women, we recognize that having measured estrogen levels or ovarian function, or having recorded the use of estrogens or hormone therapy, in our patients would have been of interest.

The fact that ApoC3 is related to the activity, severity, and damage produced by the disease, and that, besides, this molecule has been associated with CV disease in the general population, is of great interest. We hypothesize that ApoC3 could be a pathogenic mechanism that links the damage caused by the disease with the CV disease that these patients express. Prospective studies studying the relationship between ApoC3 and subclinical CV disease or cardiovascular events in patients with SLE will be required in the future.

## Conclusion

In conclusion, the activity and severity of SLE, and the damage accumulated by patients with this disease, are associated with higher levels of ApoC3.

## Supplementary Information

**Additional file 1:** **Supplementary Table 1.** Multivariable analysis of the relationship of SLE activity, severity, and damage scores with apolipoprotein C3. Patients stratified in different categories.

## Data Availability

The data sets used and/or analyzed in the present study are available from the corresponding author upon request.

## Abbreviations

ACA: Anticardiolipin antibodies
ANA: Antinuclear antibodies
ApoC3: Apolipoprotein C-3
BMI: Body mass index
CI: Confidence of interval
CRP: C-reactive protein
CV: Cardiovascular
DMARD: Disease-modifying antirheumatic drug
ELISA: Enzyme-linked immunosorbent assay
ENA: Extractible nuclear antibodies
HDL: High-density lipoprotein
IQR: Interquartile range
LDL: Low-density lipoprotein
SD: Standard deviation
SLE: Systemic lupus erythematosus
SLEDAI: Systemic Lupus Erythematosus Disease Activity Index
SLICC: Systemic Lupus International Collaborating Clinics Damage Index
