# Supplementary material for: Apolipoprotein C-III in patients with systemic lupus erythematosus
Source: Arthritis Res Ther. 2022 May 10;24:104. doi: 10.1186/s13075-022-02793-y (PMC9088095; doi:10.1186/s13075-022-02793-y)
Supplement: Supplementary file 1 — Additional file 1: Supplementary Table 1. Multivariable analysis of the relationship of SLE activity, severity, and damage scores with apolipoprotein C3. Patients stratified in different categories. [file 13075_2022_2793_MOESM1_ESM.docx]

| **Supplementary Table 1. Multivariable analysis of the relationship of SLE activity, severity, and damage scores with apolipoprotein C3. Patients stratified in different categories.** | | | | | | | | | |  |
| --- | --- | --- | --- | --- | --- | --- | --- | --- | --- | --- |
|  | SLICC $\geq$1 | SLEDAI $\geq$1 |  | SLEDAI categories | | | | | | |
|  |  |  |  | No activity | Mild or Moderate | | | High or Very High | |  |
|  | Beta coef. (95%CI), p | | | | | | | | | |
| Lipid profile |  |  |  |  |  | | |  | |  |
| Cholesterol, mg/dl | 5 (-8-17), 0.47 | -3 (-14-8), 0.56 |  | ref. | -4 (-15-8), 0.54 | | | -1 (-23-21), 0.91 | |  |
| Triglycerides, mg/dl | 16 (-11-43), 0.25 | -3 (-28-21), 0.80 |  | ref. | -3 (-28-23), 0.83 | | | -6 (-55-42), 0.80 | |  |
| HDL cholesterol, mg/dl | 0 (-7-7), 0.94 | -1 (-8-5), 0.65 |  | ref. | -2 (-8-5), 0.56 | | | 2 (-10-14), 0.75 | |  |
| LDL cholesterol, mg/dl | 1 (-8-11), 0.82 | -1 (-10-7), 0.77 |  | ref. | -1 (-10-8), 0.80 | | | -2 (-19-15), 0.81 | |  |
| LDL: HDL cholesterol ratio | 0.01 (-0.24-0.26), 0.97 | 0.03 (-0.19-0.25), 0.79 |  | ref. | 0.05 (-0.18-0.28), 0.67 | | | -0.11 (-0.55-0.33), 0.64 | |  |
| Non-HDL cholesterol, mg/dl | 5 (-8-17), 0.47 | -3 (-14-8), 0.55 |  | ref. | -4 (-15-8), 0.53 | | | -1 (-23-21), 0.92 | |  |
| Apolipoprotein A1, mg/dl | 3 (-9-16), 0.60 | -1 (-13-10), 0.81 |  | ref. | -3 (-15-9), 0.62 | | | 9 (-13-31), 0.42 | |  |
| Apolipoprotein B, mg/dl | -2 (-9-6), 0.70 | -3 (-10-5), 0.42 |  | ref. | -3 (-10-4), 0.41 | | | -2 (-15-12), 0.79 | |  |
| Apo B: A1 ratio | -0.02 (-0.07-0.04), 0.56 | 0.00 (-0.05-0.05), 0.88 |  | ref. | 0.00 (-0.05-0.05), 0.93 | | | -0.01 (-0.11-0.08), 0.78 | |  |
| Lipoprotein (a), mg/dl | 2 (-28-33), 0.88 | 2 (-25-29), 0.88 |  | ref. | 0 (-28-28), 0.97 | | | 13 (-40-67), 0.62 | |  |
| Atherogenic index | 0.10 (3.04-3.70), 0.60 | 0.01 (-0.32-0.34), 0.96 |  | ref. | 0.04 (-0.30-0.37), 0.83 | | | -0.19 (-0.83-0.46), 0.57 | |  |
| Apolipoprotein C3 |  |  |  | ref. |  | | |  | |  |
| Unadjusted | **0.46 (0.07-0.85), 0.020** | **0.43 (0.08-0.78), 0.017** |  | ref. | **0.39 (0.03-0.75), 0.035** | | | **0.69 (0.02-1.37), 0.045** | |  |
| Adjusted #1 | 0.23 (-0.16-0.62), 0.24 | **0.42 (0.09-0.75), 0.014** |  | ref. | **0.36 (0.03-0.70), 0.035** | | | **0.86 (0.20-1.52), 0.011** | |  |
| Beta coefficients consider apolipoprotein C3 as the dependent variable. | | |  |  |  | | |  | |  |
| LDL: low-density lipoprotein; HDL: high-density lipoprotein. | | |  |  |  | | |  | |  |
| SLEDAI: Systemic Lupus Erythematosus Disease Activity Index. | | |  |  |  | | |  | |  |
| SLEDAI categories were defined as: 0, no activity; 1-5 mild; 6-10 moderate; >10 activity. | | | | |  | | |  | |  |
| SLICC: Systemic Lupus International Collaborating Clinics/American Colleague of Rheumatology Damage Index. | | | | | | | |  | |  |
| #1 adjusted for age, BMI, systolic blood pressure and statins.  No further adjustment for lipid profile molecules was necessary since none of them had a relation with the scores with a p value inferior to 0.20. | | | | | |  |  | |  |  |
